# Urban and Home‐Based Vegetable Gardening Practices in Ethiopia: A Review on Challenges, Opportunities, and Perspectives

**DOI:** 10.1155/tswj/6484062

**Published:** 2026-08-03

**Authors:** Mengistu Wubie Birhanu, Zebyder Temesgen Negussie

**Affiliations:** ^1^ Department of Horticulture, College of Agriculture and Natural Resources, Debre Markos University, Debre Markos, Ethiopia, dmu.edu.et

**Keywords:** challenges and opportunities, nutritional security, school gardens, urban gardening, vegetable production

## Abstract

Rapid urbanization, driven by the search for better opportunities and improved living conditions, has led to rising unemployment and limited access to nutritious and affordable food. Urban and home‐based vegetable gardening has emerged as a practical strategy to address food insecurity, malnutrition, and environmental degradation in rapidly urbanizing areas. This review synthesizes evidence from peer‐reviewed articles published between 2015 and 2025 (following PRISMA protocol) to assess the status, challenges, and opportunities of home vegetable gardening in Ethiopia. It highlights key challenges, including limited access to land and clean water, gendered barriers to women, limited technical knowledge, inadequate extension services, and weak institutional support, all of which hinder production and sustainability. Moreover, the widespread use of untreated wastewater for irrigation poses significant health and environmental risks due to heavy metal contamination and causes soil‐transmitted infectious diseases. Despite these challenges, opportunities such as utilizing vacant urban lots, promoting vertical gardening, and supporting local seed production remain untapped. Furthermore, although government support for urban gardening has grown recently, its implementation remains inconsistent. This review also identifies a significant geographical gap within the literature, with gardening practices and interventions concentrated in Addis Ababa, Sidama, Amhara, and Tigray. Effective interventions should focus on expanding and scaling up, enhancing institutional capacity, strengthening extension systems, encouraging sustainable practices, and integrating urban vegetable gardening into broader food system planning. Addressing these issues can enhance the role of vegetable gardening as a vital element of resilient urban development and food system transformation.

## 1. Introduction

Urbanization is a worldwide trend transforming our planet, with around 68% of the global population projected to reside in cities by 2050 [1]. In Sub‐Saharan Africa (SSA), about 42% of the population lives in urban areas, increasing urban population growth, food prices, and unemployment [2]. The Global Hunger Index report indicates that SSA faces the highest levels of food insecurity, with 27.4% of the population undernourished and a notable rate of child stunting [3]. Additionally, nearly 90% of urban population growth is occurring in Africa and Asia, with Ethiopia undergoing the fastest urbanization in SSA [4]. Ethiopia′s urban population increased from 21.6 million in 2017 to 25 million by 2020, driven by rural–urban migration, settlement reclassification, and natural growth [5]. This rapid urban expansion leads to rising urban poverty, loss of agricultural land, and a shift toward processed and imported foods, which affect dietary habits. Severe acute malnutrition remains a significant challenge, particularly for children under five, highlighting the urgent need for alternative urban food production systems and livelihood opportunities [6, 7].

Urban agriculture (UA) emerges as a strategy to improve food security and reduce urban poverty in rapidly growing cities [8]. It encompasses various agricultural activities such as apiculture, aquaculture, poultry farming, livestock rearing, and the cultivation of crops like fruits, vegetables, mushrooms, and medicinal plants in urban or periurban areas [9, 10]. UA promotes a sustainable and resilient food system, enhancing access to fresh, healthy food, reducing food miles and carbon footprints, improving urban biodiversity, creating green spaces, fostering social inclusion, and supporting local economies [11]. Urban gardening is the most common form of UA, involving crop cultivation in urban environments, and has gained attention in SSA for its potential to improve food security, nutrition, and environmental sustainability [10, 11].

Urban gardening has significant potential to address both unemployment and food insecurity, providing solutions to the challenges caused by rapid urbanization. Despite a growing research interest, previous reviews related to home gardening in Ethiopia primarily focused on rural areas, emphasizing ethnobotanical and agroforestry systems. Moreover, research on UA is predominantly centered in big cities like Addis Ababa [11], with initiatives like Green Legacy promoting fruit farming [12, 13]. This has led to a limited understanding of how gardening practices adapt to rapidly expanding urban cities/towns with limited space across the country. Identifying knowledge gaps and generating evidence on home vegetable gardening are crucial for creating targeted interventions that enhance nutritional outcomes. This review addresses urban vegetable gardening practices, dietary diversity, nutrition outcomes, crop diversity, gender roles, as well as major challenges and opportunities associated with urban vegetable gardening. The review has the potential to inform future research priorities and guide the development of policies and interventions that strengthen sustainable food production systems in urban areas.

## 2. Review Methodology

The articles for this review were sourced from three electronic databases: PubMed, Scopus, and Google Scholar. To include the most recent evidence and policy changes, we focused on articles published between January 1, 2015, and December 31, 2025. A comprehensive search strategy was employed, utilizing Medical Subject Headings (MeSH), indexed keywords, and free‐text terms with Boolean operators (OR, AND) to connect four key concepts: Geography in Ethiopia, activities such as UA, home gardening, and homestead production, outcomes like vegetable production, food security, and nutrition, and socioeconomic factors such as gender, women′s empowerment, and barriers to land access.

Each database had tailored search strategies. For PubMed, we used the terms (Ethiopia [tiab] OR Ethiopia [Mesh]) combined with (Urban Agriculture [MeSH] OR garden ^∗^[tiab] OR homestead ^∗^[tiab] OR urban farm ^∗^[tiab] OR periurban [tiab]) and (vegetable ^∗^[tiab] OR Vegetables [MeSH] OR horticulture [tiab] OR food security [tiab] OR nutrition sensitive [tiab] OR crop productivity [tiab]). In Scopus, the search terms included TITLE‐ABS (Ethiopia) and TITLE‐ABS‐KEY (“urban agriculture ^∗^” OR “garden ^∗^” OR “homestead ^∗^” OR “kitchen garden ^∗^” OR “peri‐urban”), AND TITLE‐ABS‐KEY (“vegetable ^∗^” OR “horticulture” OR “food security” OR “nutrition sensitive” OR “crop productivity”). Google Scholar searches used keywords such as “Ethiopia,” “home garden,” “urban agriculture,” “homestead,” “women,” “gender,” “female land,” “barriers,” and “constraints.” All results were imported into Zotero for deduplication.

**Figure 1:**
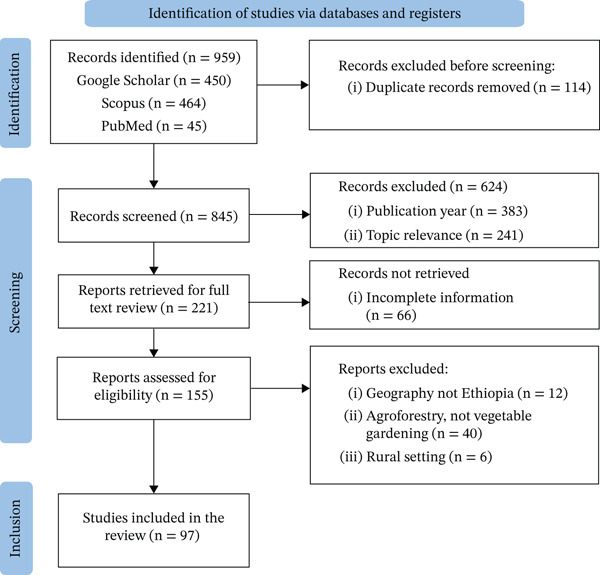
Flowchart for literature selection process. *Source:* Authors′ compilation (2026) following PRISMA protocol.

A rigorous double‐blind screening process was employed, allowing only peer‐reviewed primary research, systematic reviews, and relevant English‐language reports on Ethiopian home and urban gardening, particularly with a gendered perspective. Conference abstracts lacking full texts or primary data on Ethiopia were excluded. Additionally, literature on UA trends in SSA was considered for context, along with reports and policies from key governmental and nongovernmental organizations. The selection process followed PRISMA criteria, which included identification, screening, eligibility assessment, and inclusion (Figure 1). From an initial review of 959 articles, 97 were found to be relevant and aligned with the objectives of this study.

## 3. UA: An Overview

UA has been practiced for centuries, with evidence dating back to ancient civilizations like Mesopotamia and Egypt [14]. In these societies, agriculture was integrated into city life, supporting local food production within urban centers. Historically, UA has been vital during periods of food scarcity and crises. The cultivation of underutilized indigenous leafy vegetables holds significant promise for enhancing household food security and generating income [15]. Over the 20th century, UA evolved from traditional practices to modern approaches, gaining recognition for its role in enhancing food security, promoting sustainability, and encouraging community gardening, especially in SSA [9, 16].

In SSA, many urban households rely on UA as a source of income and food for household consumption, thereby contributing to improved food and nutritional security among low‐income residents [16, 17]. Some researchers argue that UA should be viewed as a coping mechanism rather than a comprehensive solution to food insecurity [18, 2, 17]. Nevertheless, strong support from policymakers is crucial to integrating UA into broader development strategies, which could significantly boost food security for urban households [19].

The role of UA as a buffer for food security and urban resilience has become pronounced during the COVID‐19 pandemic, environmental disasters, and conflicts [20–23]. Lockdowns and supply chain disruptions during COVID‐19 renewed interest in UA as a safeguard against food shortages. Furthermore, during periods of isolation, community gardens and urban farming projects provided benefits beyond food; they supported mental health, social interaction, and community solidarity [19]. Previous research has highlighted the positive impact of gardening activities on mental health and quality of life [24]. Fresh, locally grown food became vital for survival during harsh conditions and conflicts [17, 22]. As a result, urban horticulture, particularly vegetable gardening, is experiencing significant growth not only in SSA but also worldwide, engaging over 100 million people due to its paramount role in global food security [25].

Currently, UA is considered a key component of climate‐smart agriculture, enhancing productivity and fostering sustainability amid climate change by reducing the use of synthetic chemicals, promoting environmental sustainability, and enhancing healthy food production [26, 20]. More than 20% of urban households in low‐ and middle‐income countries are engaged in UA. Although UA is expanding across SSA, its potential contribution to sustainable urban food production is limited by numerous socioeconomic and institutional challenges. Rapid urbanization, high population density, land scarcity, limited access to irrigation, and inadequate policy and institutional support undermine its effectiveness [14, 15]. For example, a study in Kenya and Zambia found that 33% of households participate in UA, primarily due to informal settlements, land tenure, and proximity to food retailers [17]. Additionally, the use of contaminated wastewater for irrigation poses significant environmental and public health risks, leading to soil pollution and the potential spread of harmful pathogens [27]. Addressing these issues requires a well‐planned, locally driven, multisectoral approach that incorporates innovative technologies [11, 25, 28].

## 4. Home Vegetable Gardening, Dietary Diversity, and Nutrition Outcomes in Ethiopia

### 4.1. Urban Vegetable Gardening

Rapid urbanization in Ethiopia has intensified several socioeconomic challenges such as food insecurity, unemployment, climate change, and public health, particularly among low‐income urban communities. As a result, urban gardening has become an important component of Ethiopia′s policy and development agenda [11]. For example, the government is actively encouraging home gardening to boost the production of fruits and vegetables, including leafy vegetables, for household consumption and income [29]. Additionally, planting fruit trees is a major focus of the country′s green legacy initiatives, launched in 2019, which aim to restore the ecology and protect biodiversity [12].

Few nongovernmental organizations (NGOs) and international development partners are involved in urban gardening in Ethiopia. For instance, the Global Initiative for Sustainable Development (GIZ), the Food and Agriculture Organization (FAO), and World Vision have provided financial support to low‐income communities following the training program on urban greening, composting, and rooftop gardening [11]. Furthermore, the Ethiopian Horticulture Producer Exporters Association (EHPEA) has recently initiated urban vertical farming as a practical, scalable solution to produce fresh vegetables. The initiative aimed to equip urban youth and women with technical and business skills through training in hydroponics and other protected cultivation technologies. Such programs highlight the promising potential of hydroponic and floating systems to supply vegetables year‐round in cities with limited land. Additionally, community‐based vertical gardening projects in urban areas use vegetable bags, or “grow bags,” to cultivate leafy vegetables [30]. These initiatives have strengthened local capacities and improved the overall effectiveness of urban development and gardening.

**Figure 2:**
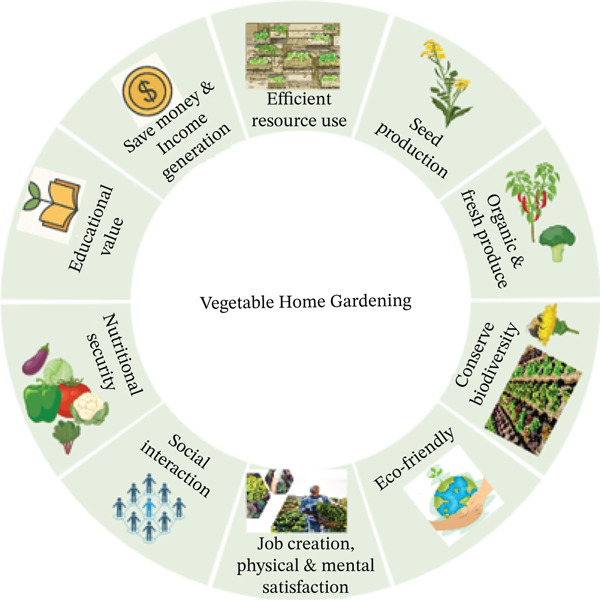
The multidimensional role of urban vegetable gardening on livelihood enhancement, environmental sustainability, and physical and mental health. *Source:* Authors′ compilation using http://Biorender.com.

Currently, integrating UA with green infrastructure is an emerging strategy to improve urban resilience and environmental sustainability [31]. In Addis Ababa, a huge urban greening project has been planned in collaboration with China to provide ecological, social, and economic benefits, including recreation, improved physical and mental health, and ecosystem regulation [32]. In this context, the growing recognition of urban gardening′s role in ecosystem services, such as microclimate regulation, urban greening, and waste recycling, provides a significant opportunity to integrate it into the urban governance framework. Composting and organic waste from households and dining facilities have great potential to promote recycling organic waste and enhance soil fertility in urban areas. Furthermore, urban gardening maximizes limited residential space and promotes on‐farm biodiversity by preserving diverse plant species and local landraces. Beyond economic and environmental benefits, urban gardening also significantly improves livelihoods and overall human well‐being (Figure 2).

**Figure 3:**
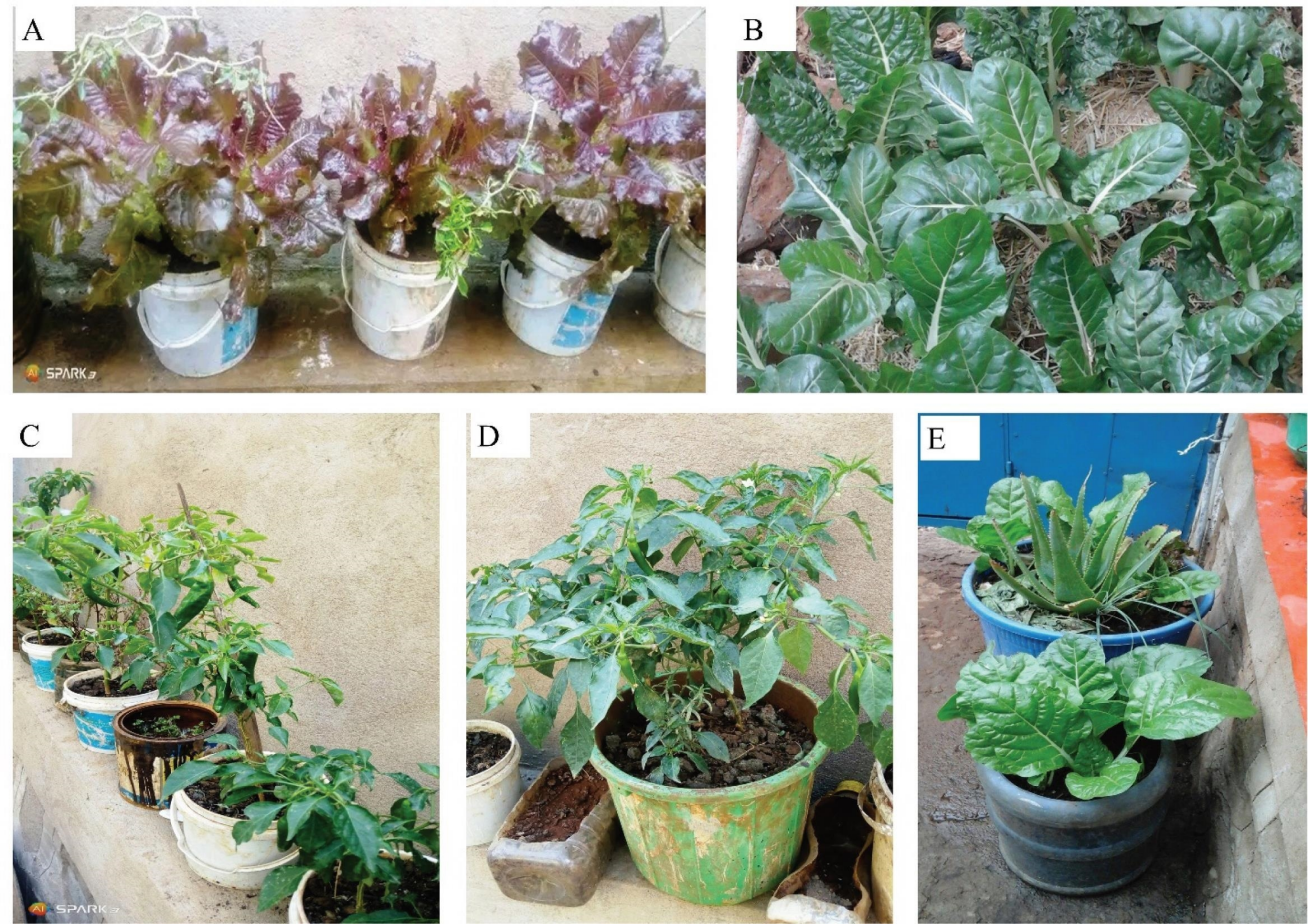
Vegetable gardening practices in space‐constrained urban centers using plastic containers. (A) *Lactuca sativa*, (B and E) *Beta vulgaris*, and (C and D) *Capsicum annuum*. *Source:* photos taken by the author during a field visit to Debre Markos City, Northwest Ethiopia.

Urban vegetable gardening is becoming more common across different regions and cities in Ethiopia (Table 1). In this regard, cultivating vegetables within household compounds and other underutilized urban spaces has become an accessible and practical approach for urban residents (Figure 3). In Addis Ababa, urban vegetable gardening primarily focuses on leafy greens and other nutrient‐dense crops, such as Swiss chard, cabbage, beetroot, and carrots [33, 34]. Households benefit from improved access to fresh, nutritious foods and reduced grocery expenses, which helps to enhance overall food security and dietary quality, despite its limitations (see Section 7.1). However, traditional backyard gardening in rapidly growing cities like Addis Ababa and Bahir Dar is often constrained by limited residential space [31]. To overcome these challenges, adopting innovative gardening approaches, such as container gardening and vertical farming, enables multiple layers of crops in confined spaces, effectively increasing planting density without requiring additional land. Such practices are especially applicable for leafy and other high‐value vegetable crops that are regularly consumed and sold within urban areas.

**Table 1:** Urban home vegetable gardening practices across cities and towns in Ethiopia.

Home gardening activity	Location (city/town)	Crop species	Major contribution	Source
Vertical farming (stacked beds)	Sidama Region, South Ethiopia	Tomato (*Solanum lycopersicum*)	Vertical systems with optimized vertical and horizontal spacing increased productivity per unit area.	[35]
Container and multilayer gardening	Bahir Dar	Leafy vegetables	Container and pot gardening increases access to vegetables and enhances household nutritional outcomes.	[36]
Riverbank garden plots (informal)	Addis Ababa	Leafy vegetables and herbs	Nutritional and food security benefits exist, but water quality is a major concern.	[37]
Greywater‐fed vertical and keyhole gardens	Different towns	Various vegetables	The integration of greywater with vertical and keyhole systems enhanced water use efficiency through careful management to prevent contamination.	[30]
Permagarden	Different regional cities and towns	Fruits and vegetables	Home gardening increased the frequency and diversity of vegetable and fruit consumption among vulnerable households.	[38]
Container/balcony gardening	Bahir Dar	Tomatoes, kale, and lettuce	Vegetable production throughout the year in a limited space.	[39]

Most commonly, vegetables are grown on balconies and in vacant areas within residents′ compounds using gardening containers, including growing bags, recycled containers, plastic pots, and shelves [39]. Our observations from Debre Markos city confirmed that old plastic containers and boxes are reused as effective gardening tools for growing vegetables such as *Lactuca sativa*, *Beta vulgaris*, and *Capsicum annuum* (Figure 3 **)**. Additionally, a study in Sidama region demonstrated that multilevel vertical farming structures significantly increased tomato yield per unit area [35]. The researcher noted that optimizing both horizontal and vertical spacing enhanced fruit production and economic returns, indicating that vertical farming can be applied practically in urban horticulture.

### 4.2. Urban Food Systems and Dietary Diversity

**Figure 4:**
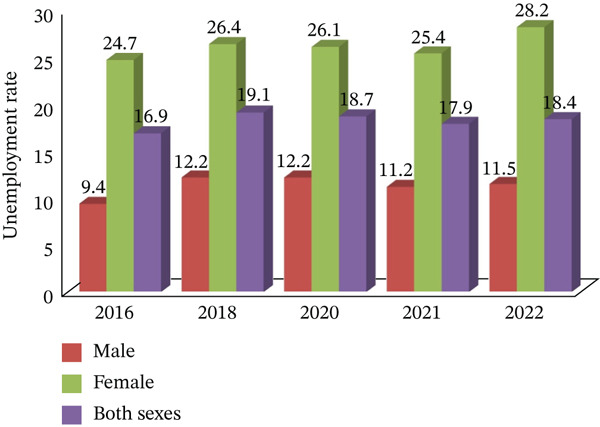
Trends in the unemployment rate among individuals aged 10 years and older in urban areas of Ethiopia. *Source:* adopted from UEUS [40].

The food system in general presents a significant challenge to urban development in SSA, linked to unemployment, poverty, and poor health outcomes [2]. Unemployment among young people is a growing concern. For instance, the urban unemployment rate in Ethiopia increased by 2.5% over 5 years, from 16.9% in 2016 to 19.4% in 2022 [40] (Figure 4). As a result, urban food production has gained increasing attention, particularly for its potential to diversify employment and income sources, improve dietary quality, and increase waste recycling in urban households, thereby promoting sustainable urban development [41].

The Ethiopian diet is predominantly cereal‐based, with limited consumption of fruits and vegetables. A fermented flatbread (*Injera)* made from cereals including *Triticum aestivum*, *Zea mays*, and *Eragrostis tef* is the most popular cuisine in both urban and rural areas. However, such a monotonous dietary patternis insufficient to meet micronutrient needs [42]. The cultural and socioeconomic factors substantially influence such dietary habits [39, 34]. On the other hand, there is a growing preference for animal‐based and processed foods, along with a willingness to pay for them [43]. Nevertheless, processed foods available in supermarkets are mostly accessible to middle‐ and high‐income urban residents who can afford the higher prices [44]. Additionally, high prices for fruits and vegetables, especially during certain seasons, affect purchasing decisions. For example, during fasting periods in Ethiopian Orthodox Christianity, when followers avoid consuming animal products, vegetable prices tend to rise significantly [45].

Generally, the combined impact of low dietary diversity and increased consumption of processed foods has negative health outcomes. A low‐diversity diet is typically associated with poor dietary quality, following limited intake of nutrient‐rich vegetables and other essential food groups [46]. In urban areas, obesity among adult women has reached 21%, whereas stunting remains prevalent among children under 5 years old [47]. Meanwhile, research in Addis Ababa indicated that adolescent girls are at a higher risk of poor dietary diversity, possibly because of limited access to nutrition education and less autonomy over food choices [48].

### 4.3. Home Vegetable Gardening and Nutrition Outcomes

**Figure 5:**
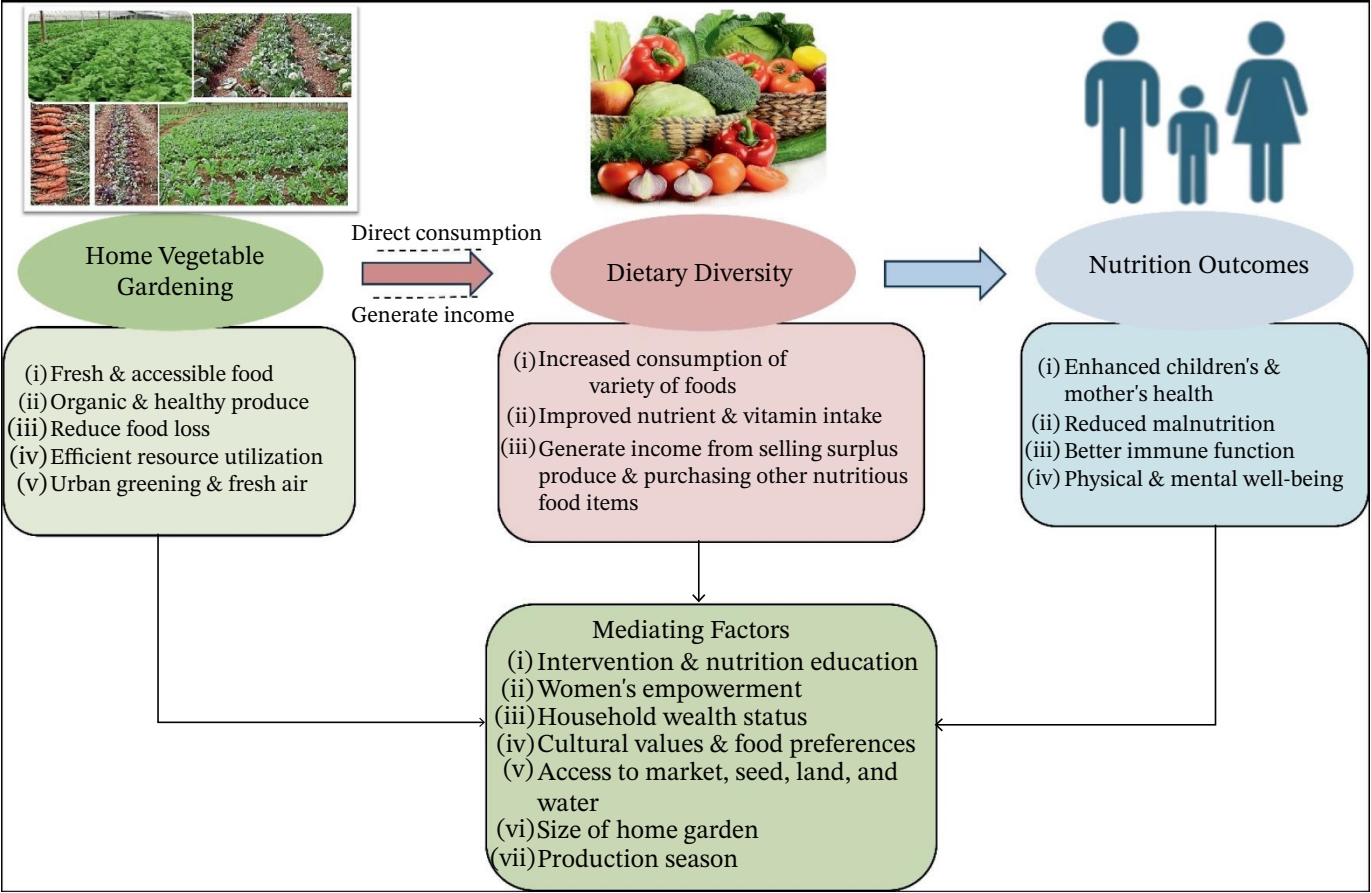
Conceptual schematic framework on home vegetable gardening, dietary diversity, and nutrition outcomes as influenced by socioeconomic and cultural factors.

Engaging in year‐round home vegetable gardening increases vegetable consumption while also sustaining dietary diversity (Figure 5). Moreover, dietary diversity can be improved by selling surplus vegetables and reducing the cost of purchasing vegetable items. Research in the Amhara region of Northwest Ethiopia shows that households with home gardens experience significantly higher food security than those without [49]. Production and consumption of vegetables increase vitamin intake and dietary diversity, reducing the risk of micronutrient deficiency [46]. Vegetables provide antioxidants and are rich sources of essential minerals such as Fe, Zn, Ca, K, and P [50]. As a result, home‐grown green vegetables are frequently preferred over market‐bought alternatives due to their freshness and less contamination.

Interventions involving gardening activities significantly increased vegetable and fruit consumption [38]. This suggests that nutrition education programs at schools, community centers, and healthcare facilities could improve health and promote healthier dietary choices within the community [51]. For example, due to nutrition education and home gardening efforts in the Jimma Zone, Southwest Ethiopia, there was a significant increase in average birth weight and maternal hemoglobin levels [52]. Similarly, vegetable production and crop diversification through home gardening are promising strategies to improve food and nutrition security in Southern Tigray [53]. Despite these positive associations, reports on the effect of vegetable gardening on child stunting are not always conclusive. For example, a comparative study in the Amhara region, Northwest Ethiopia, reported that the nutritional status of children aged 6–59 months did not differ significantly between households with and without home gardens [54]. This highlights the complexity of nutritional outcomes and the influence of socioeconomic and health factors. Additionally, home gardens also provide therapeutic benefits for women living with HIV [39]. They have been shown to improve mental health, reduce stress, and foster a sense of purpose and achievement, thereby boosting self‐esteem and resilience.

## 5. Crop Diversity and Species Composition in Home Gardens

Crop species diversity in home gardens plays a crucial role in ensuring household food security and contributes to environmental sustainability through on‐farm biodiversity conservation [55, 56]. A wide variety of vegetables are commonly grown in home gardens, including Swiss chard, kale, carrots, head cabbage, potatoes, lettuce, green peppers, garlic, onions, shallots, beetroot, tomatoes, and cucumbers [57–60]. These crops are mainly grown for home consumption to reduce reliance on market purchases, as the availability and prices of fruits and vegetables are often unstable.

Home gardens in the Southern region of Ethiopia are well known for their high species diversity, including food, medicinal, and ornamental crops [61]. Specifically, Enset (*Ensete ventricosum*)‐based home gardens serve as a sustainable land‐use system that integrates biodiversity conservation, livelihood support, and climate‐resilient food production in semiarid areas [62]. Similarly, in Northwest Ethiopia, home gardens harbor a variety of vegetables, fruits, cereals, legumes, spices, and other species [63]. In Bahir Dar city, home gardens exhibit high crop diversity, including mango, avocado, papaya, head cabbage, tomato, and lettuce [56]. The preservation of this diverse array of crop varieties is supported by a combination of agroecology, traditional practices, cultural significance, and the cultural values associated with the crop species [64].

A recent study in the Oromia region revealed a high plant species diversity in home gardens, including herbs, multipurpose trees, shrubs, and climbers [65]. *Allium sativum, Vicia faba, Z. mays, Solanum tuberosum*, and *Brassica oleracea* are food crops that account for about 31% of the total diversity. Medicinal plants, including *Ruta chalepensis, Lippia abyssinica,* and *Rhamnus prinoides,* are also contained in that home garden. However, traditional and diverse home garden systems are increasingly being replaced by khat (*Catha edulis*) monoculture in some parts of the country [66]. Besides, the diversity of plant species in a home garden is influenced by the garden size, the surrounding landscape, economic, and educational levels of the households [67].

## 6. Gender Roles in Production, Management, and Utilization of Vegetable Garden

The decision‐making process for home‐based vegetable gardening is often influenced by the head of household. Usually, female‐headed households prioritize vegetables and root crops for home consumption, whereas male‐headed households are more inclined toward cultivating cereals, pulses, cash crops, and forage [68]. Women‐managed gardens often exhibit greater crop diversity in both use and species per unit area, and the produce is mainly for household consumption [57]. Herbaceous plants account for around 90% of produced home garden crops, and women preferred them.

**Figure 6:**
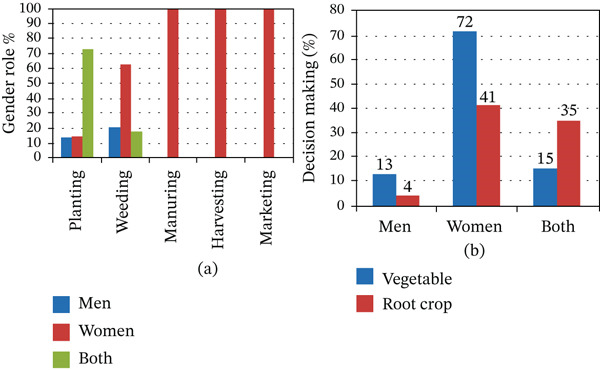
(a) Gender roles in vegetable home garden management activities and (b) decision‐making on crop type. *Source:* adopted from Abebe and Mulu [68] and Berhanu and Guye [69].

Despite cultural barriers and weak enforcement of legal frameworks constraints (see Section 7), studies conducted across different regions of Ethiopia revealed the crucial and dominant role of women in home garden management [57, 69]. This is partly because they stay home to handle household and family care [65]. In female‐headed households, women are solely responsible for all aspects of home garden management, including land preparation, seed preservation, planting, and crop care [68]. Tasks such as manuring, harvesting, and marketing are primarily carried out by women, regardless of household type (Figure 6a) [69]. Women play a vital role in conserving a wide variety of crops in home gardens, including fruits, vegetables, medicinal plants, and spices. They are also the primary decision‐makers regarding the consumption and marketing of vegetables and root crops, regardless of who heads the household (Figure 6b). Whereas, men are mainly responsible for fencing, marketing, and determining income from surplus produce [57].

## 7. Major Challenges and Opportunities of Urban Vegetable Gardening in Ethiopia

### 7.1. Challenges

Despite its potential, urban home vegetable gardening remains an underutilized economic sector, largely for subsistence purposes [41]. Perceptions of stakeholders and involvement of the private sector in vegetable gardening are also limited; often considered “The business of the poor.” [59]. Only 4.09% of wealthy, 7.12% of middle‐income, and 12.7% of low‐income households participate in urban home gardening [55]. Variability in seasonal rainfall and rising temperatures also threaten sustainable urban vegetable production, especially for stress‐sensitive leafy vegetables [36]. Other factors, such as access to land, water, seed quality, technical knowledge, and extension services, affect vegetable gardening [56].

#### 7.1.1. Gendered Constraints to Home Vegetable Gardening

Ethiopian land policy acknowledges women′s equal rights to land ownership, use, and administration. However, cultural norms and ineffective legal enforcement often hinder women′s ability to acquire, access, and manage land [70]. These challenges not only limit their access to credit but also constrain their ability to make independent decisions and invest in long‐term agricultural projects, particularly in rural areas.

In urban settings, women face significant obstacles related to labor and time, largely due to their disproportionate responsibilities for childcare and household chores. A lack of household support, combined with an increased domestic workload, restricts their capacity to engage in and expand vegetable gardening. Moreover, various socioeconomic factors, such as limited access to agricultural inputs and training services, further diminish women′s motivation and ability to adopt improved gardening techniques [71, 72].

Despite their crucial role in food security through home gardening practices, women′s contributions are often unrecognized by communities, academic circles, and policymakers [73]. Additionally, sociocultural expectations place further burdens on women, making them primarily responsible for domestic tasks. They are also more susceptible to market fluctuations because of the informal market structure and inadequate infrastructure for purchasing inputs and selling their produce [74].

#### 7.1.2. Limited Access to Inputs and Resources

**Figure 7:**
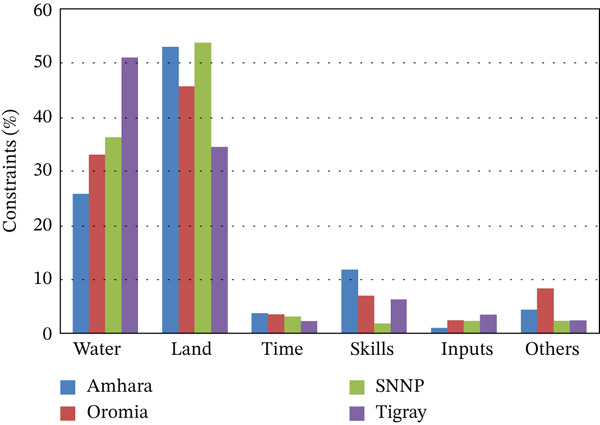
Factors affecting households′ adoption of home gardening across various regions of Ethiopia. *Source:* adopted from Hirvonen and Headey [75].

Several factors, including limited access to water sources and land, as well as a lack of input, pose significant challenges to vegetable gardening. However, the severity of these challenges varies across regions in Ethiopia (Figure 7) [75]. In Mekelle City, Northern Ethiopia, the small size of the garden and water scarcity are the major barriers to home vegetable gardening [76]. To address water scarcity, efficient water management strategies, such as rainwater harvesting or drip irrigation, are crucial.Whereas land scarcity is a common issue in densely populated and rainfall‐abundant areas such as the Southern Nations, Nationalities, and Peoples (SNNP) of Ethiopia [36]. Limited access to land restricts the cultivation of a variety of vegetables and highlights the need for innovative solutions. This can be addressed through vertical gardening techniques, implemented outdoors, on rooftops, balconies, and in containers. Besides intensive production methods, many vacant urban lots in cities and towns are often filled with solid waste and garbage [59]. The map analysis study showed that over 57% of bare land in urban and periurban areas is generally suitable for urban food production, with up to 88% suitable for growing vegetables [33]. Vacant spaces along rivers and roads can be transformed into community gardens, especially in residential areas. Turning these spaces into food production sites can support sustainable development by improving food access, increasing green infrastructure, protecting the environment, and fostering social cohesion.

#### 7.1.3. Health Risks of Wastewater Irrigation for Home Gardening

Despite its potential for irrigation and nutrient sources, untreated wastewater introduces pathogens, heavy metals, and persistent chemicals that threaten food safety and environmental health. There are concerns about greywater quality and river pollution from domestic and industrial waste discharges [58, 77]. Approximately 61% of all vegetables and 90% of leafy vegetables (e.g., lettuce, Swiss chard, and cabbage) in the city are irrigated with wastewater along the riverbanks. The main sources of wastewater in Addis Ababa and other towns are reported to be manufacturing industries near rivers that release harmful chemicals [59].

Irrigation with untreated wastewater led to soil‐transmitted helminth infections and contamination with heavy metals. In Addis Abeba, helminths such as *Ascaris lumbricoides,* hookworm, and *Trichuris trichiura* were found in 67.5% of vegetables grown with untreated wastewater and 20.8% of the stools of female farmers [37]. Furthermore, research indicates that using wastewater for vegetable cultivation contributes to heavy metal contamination in various cities and towns [78–81]. Major contaminants include lead (Pb), cadmium (Cd), mercury (Hg), and arsenic (As) [78, 82]. Several rivers flowing through the capital and regional cities serve as dumping sites for both solid and liquid waste. Studies have assessed the concentrations of Pb, Cd, Cr, Ni, and Cu in soil, vegetables, and water around Bole Lemi Industrial Park in Ethiopia, confirming that these levels exceeded the country′s regulatory limits [83]. This issue may persist unless interventions are implemented, such as deworming programs and comprehensive health education on food washing and sanitation practices during the production and consumption of vegetables. Additionally, prohibiting or regulating untreated wastewater for irrigating vegetables that are consumed raw has also been proposed as an alternative intervention to reduce microbial contamination and associated health risks [37].

#### 7.1.4. Climatic and Biological Factors

Despite the diverse and generally favorable agroecological conditions for gardening in many parts of the country, the productivity and sustainability of urban vegetable gardening are still limited by various biotic and abiotic factors [36, 56]. Climate variability is one of the major threats affecting urban vegetable production and household income [36]. Lack of early warning information hinders timely responses to climate change, worsening its effects.

Plant diseases and pests are also significant limiting factors in home gardening. Bacterial soft rot, anthracnose, and leaf spot are the most common diseases affecting fruits and vegetables grown in home gardens [55]. Furthermore, animals also cause considerable damage, especially in gardens outside residential compounds. Farmers typically rely on cultural management practices, such as applying ashes and removing infected plants. Hence, proactive management through integrated pest management (IPM) strategies is indispensable.

#### 7.1.5. Limited Research and Extension Interventions

Households engaged in vegetable gardening often lack training in improved agricultural practices, pest and disease management, and postharvest handling techniques [59]. Most farmers rely on indigenous knowledge to manage their gardens. Some vegetables can be grown year‐round in certain climates, while others, like tomatoes, are limited to specific growing seasons. As a result, home gardeners may not be able to grow their preferred vegetables year‐round and will need to plan accordingly. Therefore, farmers need guidance in selecting suitable crop species based on the local climate and growing seasons.

Furthermore, farmers are not fully tapping into the potential of urban vegetable gardening for several reasons. Limited knowledge of intensive land use, inadequate urban farming skills, lack of credit facilities, insufficient access to basic agricultural inputs, and weak extension services are the major factors influencing gardening practices [84]. The farmers also have limited information on the nutritional value of vegetables, despite the important role of fruits and vegetables in health.

### 7.2. Opportunities

#### 7.2.1. Enhanced Food Security

Home vegetable gardening provides an important opportunity to boost household food security in Ethiopia by promoting self‐sufficiency through direct consumption and income generation. Cultivating various vegetables allows families and communities to access fresh, nutritious food, reducing reliance on expensive or imported produce. Access to homegrown, healthy vegetables greatly improves consumers′ nutritional security and overall health status [59] (Figure 5).

#### 7.2.2. Job Opportunity for Poor and Vulnerable Groups

Urban vegetable gardening creates job opportunities and provides a source of income. Surplus produce can be sold at local markets, supporting the livelihoods of urban residents and benefiting disadvantaged groups by providing an alternative source of income [39]. Additionally, model home gardens can serve as community learning platforms. Sharing knowledge and experience on sustainable and productive gardening techniques can promote collective learning, empowerment, and wider adoption of similar practices within the community.

#### 7.2.3. Environmental Sustainability and Waste Management

Urban vegetable gardening promotes environmental sustainability and supports efforts to adapt to and mitigate climate change [22, 34]. Utilizing available urban spaces, rooftops, balconies, and small plots can help green cities and mitigate the urban heat island effect. Moreover, growing vegetables locally reduces the carbon footprint associated with long‐distance food transportation. Green vegetation reduces air pollution, CO_2_ emissions, and dust particles in urban areas [22]. Gardeners also recognize the environmental benefits of vegetable gardening besides providing income [34].

Growers are frequently adopting sustainable soil management practices, such as applying compost, as organic produce gains popularity both in local and global markets. Compost is a key to organic farming, where synthetic pesticides and fertilizers are minimized. The increasing demand for urban compost reflects a growing interest in organic food and sustainable agriculture [85]. Despite the potential, only a small portion of organic waste is currently used in urban gardening. Several cities face sanitation and odor problems because of improper waste disposal from dairy and poultry farms [50, 59]. Hence, urban vegetable gardening potentially addresses these problems by converting waste into valuable compost. Other organic household waste—including fruit and vegetable peels, paper, tissues, and plant residues—is possibly used as compost materials, reducing reliance on synthetic fertilizers [22]. Such practices enhance biodiversity conservation and the overall sustainability of agricultural systems [86].

#### 7.2.4. Utilizing School Gardens for Education and Nutrition Interventions

**Figure 8:**
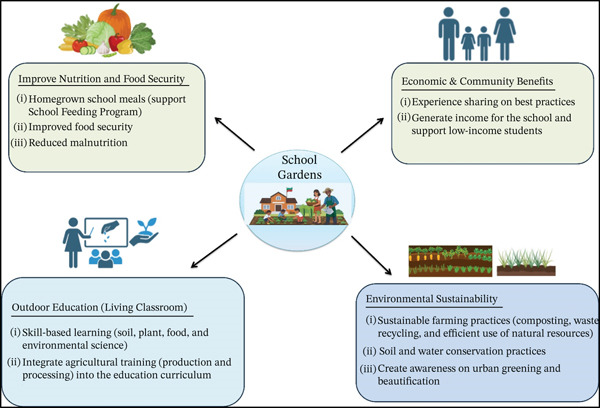
Overview of school garden initiatives in Ethiopia and their contribution to nutrition, food security, and economic benefits. The initiative also aims to improve skill‐based education by incorporating agriculture into the curriculum, promoting climate resilience and sustainable natural resource management.

Food insecurity and poor nutrition among school‐aged children remain significant challenges in Ethiopia. The government and NGOs′ policy frameworks are currently promoting the “Home Grown School Feeding Program.” The initiative aims to enhance student nutrition by providing fresh vegetables directly to schools, thereby reducing dependence on external food sources [87]. Research indicates that school feeding programs decrease dropout rates and improve academic performance [84–86]. Although school food programs are critical for immediate relief from hunger, school‐based garden education initiatives offer a sustainable alternative approach for food security and nutritional outcomes [88]. For this reason, the Ethiopian Ministry of Education has recently incorporated agriculture courses into school curricula to support long‐term food and nutrition objectives while empowering both students and communities. This educational reform is intended to integrate agriculture into students′ learning, providing practical experiences that build valuable skills (Figure 8). The initiatives also align with national sustainable development goals, emphasizing agricultural and environmental policies that focus on biodiversity conservation and agroecological management.

School gardens serve as educational demonstration sites for science, environment, climate, and nutrition (Figure 8). The abandoned land available at public educational institutions, particularly primary and secondary schools, provides excellent opportunities to establish gardens. It has been shown that school gardening practices significantly improve children′s knowledge and attitudes toward food and agricultural production. Such an approach allows students to familiarize themselves with nutritious and healthy food by integrating education, health, nutrition, and agriculture. This supports interventions that combine school gardens with nutritional education, WASH, and promotional activities, enhancing children′s awareness of vegetable cultivation and consumption, and increasing their awareness of sustainable agriculture [88–91].

#### 7.2.5. Possible Use of Treated Wastewater for Irrigation

Several rivers and small streams flow through Addis Ababa and other regional cities, providing a source of irrigation for vegetables [34, 58]. For example, large vegetable farms around Akaki and Peacock are irrigated with water from the Akaki and Bulbula rivers [82]. As mentioned above, using irrigation water from these rivers poses environmental threats and health risks because of heavy metal contamination and soil transmitting infectious diseases. To address the challenge, interventions using various techniques have emerged as alternative solutions for efficient water use in urban areas [30].

Various wastewater treatment methods are effectively employed to remove pollutants from industrial and municipal sources. Key strategies include managing pollution sources, utilizing phytoremediation, adopting cleaner technologies, and conducting regular monitoring [27]. Several methods of low‐cost wastewater treatments such as constructed wetlands, sedimentation, biofiltration, and vetiver grass (*Vetiveria zizanioides*) are practically used for the remediation of pollutants from factories and municipalities [92–94]. Employing 60 vetiver grass plants per square meter has proven effective for treating municipal wastewater in resource‐constrained urban areas due to its ability to absorb heavy metals from polluted soil [93, 94]. The combination of biofiltration and horizontal subsurface flow constructed wetlands has also been confirmed as a cost‐effective and efficient method for treating household greywater [88]. Additionally, implementing effective risk assessment and management strategies is essential for reducing exposure and ensuring crop safety in contaminated areas. In Algeria, refraining from using poorly treated wastewater led to an 85% reduction in heavy metal accumulation in vegetables [95]. If properly treated, wastewater is demonstrated as a valuable source of essential plant nutrients like phosphorus and nitrogen for urban gardening, reducing chemical fertilizer costs by 28% beyond its primary use for irrigation [96].

#### 7.2.6. Market Opportunity

Bulk vegetables sold in cities and towns are often transported from distant locations and distributed by wholesalers and retailers. Most urban gardeners grow vegetables primarily for their own use, yet there remains a significant market opportunity [34, 50]. Excess produce from vegetable gardening can be sold at the market to support household dietary needs. A major benefit of urban vegetable gardening is its ability to shorten the supply chain by reducing reliance on long‐distance transportation [22]. The income earned from selling these products can help to buy other types of food to meet household needs.

#### 7.2.7. Government and NGOs Initiatives

The Ethiopian government has prioritized the horticulture sector as demand for both fresh and processed horticultural products grows in domestic and international markets. The government supports urban vegetable gardening efforts by promoting the adoption of urban gardening practices. Despite challenges, recent initiatives by agricultural organizations and local stakeholders highlight opportunities to expand both the scale and variety of urban home vegetable gardening. In some areas, interventions provide training, improved seed varieties, and inputs to support diverse vegetable and fruit gardening systems. Hirvonen and Headey [75] evaluated how government support influenced the adoption and sustainability of homestead gardening practices within Ethiopia′s Productive Safety Net Program (PSNP) across various regions. The authors found that the program boosted the adoption of gardening practices and enhanced dietary diversity among targeted households, though regional differences affected the adoption rate. Notably, households that engaged in PSNP continued gardening after the program concluded, indicating a lasting behavioral change.

#### 7.2.8. Potential for Local Seed Production and Conservation

**Figure 9:**
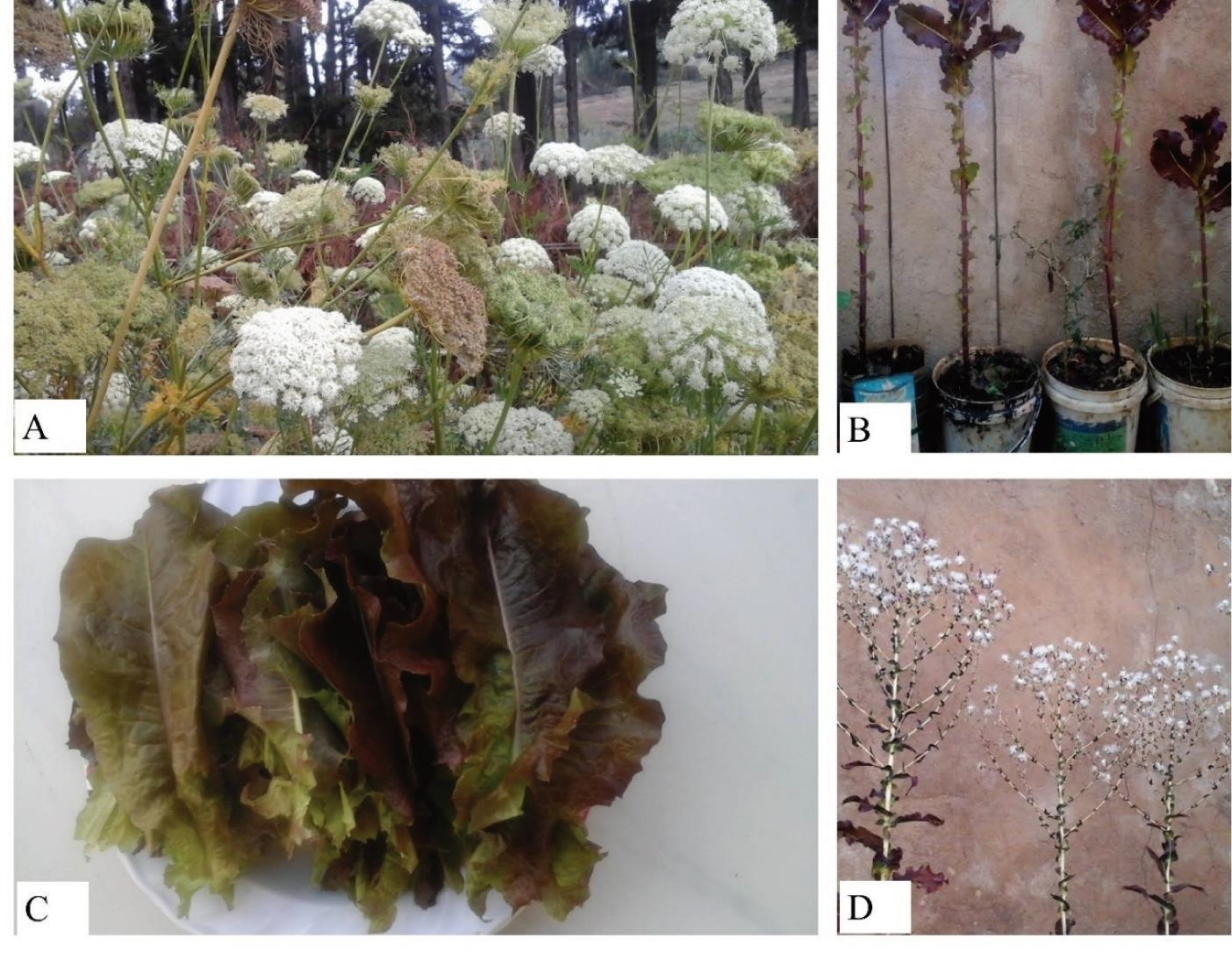
Home garden vegetable seed production practices. (A) Carrot seed production, (B and C) lettuce green leaves for consumption, (D) and seed harvest from the same planting. *Source:* photos by the author during a field visit to Debre Markos City, Northwest Ethiopia.

Vegetable production in Ethiopia faces challenges due to a shortage of high‐quality seeds, which are imported [13]. Producing vegetable seeds locally allows growers to become more self‐sufficient, reduce reliance on commercial suppliers, and ensure a steady seed supply. It has been observed that some farmers successfully produce vegetable seeds such as carrots and lettuce directly in their gardens (Figure 9A,D**)**. For certain vegetables, including Ye′abesha Gomen (collard greens) and lettuce, seed production is feasible without growing separate crops for seed and fresh consumption. Farmers selectively harvest leaves for household use while allowing the remaining plants to mature and produce seeds for later harvest (Figure 9B–D). Therefore, producing vegetable seeds in home gardens can effectively preserve indigenous varieties, which are at risk of genetic erosion [97].

## 8. Conclusions and Recommendations

Urban home vegetable gardening has enormouspotential to enhance food security, improve nutrition, and generate income, especially in low‐income and densely populated areas. School and community gardening programs also offer additional benefits by serving as platforms for environmental education, health promotion, and social bonding. One key advantage is the opportunity to access fresh, organic produce, which helps reduce consumers′ concerns regarding synthetic chemical pesticides and fertilizers used in commercial farms. To support this, urban vegetable gardens provide a practical solution by using small plots of land or vertical spaces within cities to grow a variety of vegetables. Additionally, producing vegetable seeds enables growers to become more self‐sufficient, saving money while promoting biodiversity and seed‐saving practices.

Despite the potential, urban home vegetable gardening faces various challenges, including limited access to land and water, lack of knowledge and skills, pest and disease problems, and insufficient institutional support. The widespread use of contaminated wastewater for irrigation also creates serious health and environmental risks. Farmers often rely on cultural gardening practices because of a lack of access to modern agricultural techniques and extension services. Evidence from this review demonstrates that the government, NGOs, and academic institutions have largely centered their UA practices and projects in big cities like Addis Abeba, Hawassa, Bahir Dar, and Mekelle while overlooking towns and cities in emerging regions. Expanding successful gardening practices into these underserved areas could enhance local food production and strengthen climate resilience. To overcome the challenges and foster a supportive environment for urban vegetable gardening, the government should implement the following policy measures and initiatives:

1. The Ministry of Urban Development and Infrastructure should mandate space allocation in new housing projects to promote sustainable urban vegetable gardening.
2. Municipalities should integrate urban food production into urban land‐use plans to improve access to infrastructure and production inputs, including water and land.
3. Extension services should integrate nutrition education into gardening training.
4. Government and NGOs should support women′s engagement in urban gardening through gender‐responsive training programs.

By addressing the challenges and scaling‐up effective practices, vegetable gardening can play a transformative role in developing sustainable, healthy, and resilient food systems in urban areas across the country.

## Author Contributions

M.W.B: conceptualization, investigation, writing—original draft, and visualization. Z.T.N: investigation, methodology, writing—review and editing.

## Funding

No funding was received for this manuscript.

## Disclosure

The authors have read and agreed to the final version of the manuscript.

## Ethics Statement

The authors have nothing to report.

## Conflicts of Interest

The authors declare no conflicts of interest.

## Data Availability

Data sharing not applicable to this article as no datasets were generated or analyzed during the current study.
